# Loss of Barx1 promotes hepatocellular carcinoma metastasis through up-regulating MGAT5 and MMP9 expression and indicates poor prognosis

**DOI:** 10.18632/oncotarget.18288

**Published:** 2017-05-30

**Authors:** Guodong Wang, Jian Liu, Yi Cai, Jie Chen, Wenbing Xie, Xiangqian Kong, Wenjie Huang, Hao Guo, Xiaodi Zhao, Yuanyuan Lu, Lu Niu, Xiaowei Li, Haijia Zhang, Chao Lei, Zhijie Lei, Jipeng Yin, Hao Hu, Fan Yu, Yongzhan Nie, Limin Xia, Kaichun Wu

**Affiliations:** ^1^ State Key Laboratory of Cancer Biology, National Clinical Research Center for Digestive Diseases and Xijing Hospital of Digestive Diseases, Fourth Military Medical University, Xi'an 710032, Shaanxi Province, People's Republic of China; ^2^ Department of Oncology and The Sidney Kimmel Comprehensive Cancer Center at Johns Hopkins, The Johns Hopkins University School of Medicine, Baltimore, MD 21287, USA; ^3^ Department of Orthopedic Oncology, Tangdu Hospital of Fourth Military Medical University, Xi'an 710038, Shaanxi Province, People's Republic of China; ^4^ Department of Gastroenterology, Tongji Hospital of Tongji Medical College, Huazhong University of Science and Technology, Wuhan 430030, Hubei Province, People's Republic of China; ^5^ Department of Gastroenterology, the Fifth Hospital of the People's Liberation Army, Yinchuan 750000, Ningxia Province, People's Republic of China; ^6^ State Key Laboratory of Military Stomatology & National Clinical Research Centre for Oral Diseases & Shaanxi Key Laboratory of Oral Diseases, Department of Prosthodontics, School of Stomatology, Fourth Military Medical University, Xi'an 710032, People's Republic of China

**Keywords:** hepatocellular carcinoma, barx homeobox 1, mannosyl (alpha-1,6-)-glycoprotein beta-1,6-N-acetyl-glucosaminyltransferase 5, matrix metallopeptidase 9, metastasis

## Abstract

Metastasis is the major dominant reason for poor prognosis of hepatocellular carcinoma (HCC) after surgical treatment. However, the molecular mechanism of metastasis has not been well characterzied. Here, we report a novel function of Barx homeobox1 (Barx1) in inhibiting HCC invasion and metastasis. Barx1 expression is significantly decreased in human HCC tissues than in adjacent non-tumorous tissues and normal liver tissues. Low Barx1 expression is correlated with higher tumor-nodule-metastasis stage and indicates poor prognosis. Down-regulation of Barx1 promotes HCC migration, invasion and metastasis, whereas up-regulation of Barx1 inhibits HCC migration, invasion and metastasis. Mannosyl (alpha-1,6-)-glycoprotein beta-1,6-N-acetyl-glucosaminyltransferase 5 *(MGAT5)* and matrix metallopeptidase 9 *(MMP9)* are direct target genes of Barx1. Knockdown of Barx1 up-regulates MGAT5 and MMP9 expression in HCC cells with low metastatic capability, whereas over-expression of Barx1 suppresses their expression in HCC cells with high metastatic capability. Knockdown of both MGAT5 and MMP9 significantly decreases the invasion and metastasis abilities induced by Barx1 knockdown. Barx1 expression is negatively correlated with MGAT5 and MMP9 expression in human HCC tissues. Patients with low expression of Barx1 and high expression of MGAT5 or MMP9 are associated with poorer prognosis. Thus, loss of Barx1 represents a prognostic biomarker in human HCC patients.

## INTRODUCTION

According to the WHO, hepatocellular carcinoma (HCC) is listed as the fifth most common cancer and the third most prevalent cause of cancer mortality worldwide [1]. Metastasis plays a dominant role in the high recurrence and poor survival of HCC after curative resection [2]. Although extensively investigated, the molecular mechanisms underlying the progression and metastasis of HCC remain largely unknown. Therefore, it is important to identify new potential prognostic markers and treatment targets for human HCC.

Human Barx homeobox 1 (*Barx1*) gene is located on chromosome 9q12 and belongs to the Bar class of homeobox gene family [3]. Barx1 was first isolated from a mouse embryo tissue and was identified as a repressive transcription factor which directly binds to the *NCAM* promoter and inhibits it's transcription [4]. Several studies reported that Barx1 plays important roles in cell differentiation, cell adhesion and cytoskeletal remodeling [5–8]. Barx1 shares 87% amino acid identity with its paralogue, Barx2, and both of them are ubiquitiously expressed in multiple epithelial tissues [9]. In addition, Barx2 expression was significantly down-regulated in several human cancers including HCC, and Barx2 was identified as a tumor suppressor [10–13]. Interestingly, *Barx1* gene promoter was hypermethylated in human colorectal cancer and gastric cancer, and Barx1 expression was lower in cancer tissues than adjacent noncancer tissues [14, 15]. Furthermore, over-expression of Barx1 in gastric cancer cells reduced cell proliferation [15]. These studies suggest that Barx1 may contribute to cancer progression and metastasis.

The clinicopathologic significance and potential function of Barx1 in HCC has not been reported in the literature. In this study, we report that low expression of Barx1 serves as a biomarker for poor prognosis and it associates with both HCC invasion and metastasis. Furthermore, Barx1 inhibits HCC invasion and metastasis through inhibiting *MMP9* and *MGAT5* transcription.

## RESULTS

### Low expression of Barx1 correlates with poor prognosis in human HCC

**Figure 1:**
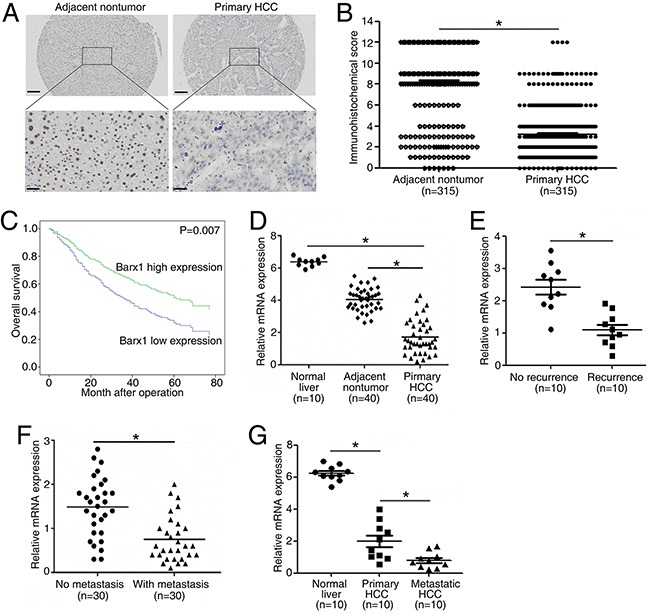
Barx1 is significantly down-regulated in human HCC tissues and low expression of Barx1 indicates poor prognosis **(A)** Representative Barx1 expression in adjacent non-tumorous tissues and primary HCC tissues detected by immunohistochemical methods. Scale bars represent 200 μm (low magnification) and 50 μm (high magnification). **(B)** Comparison of Barx1 expression in primary HCC tissues and adjacent non-tumor tissues. **(C)** Kaplan-Meier analysis of the correlation between Barx1 expression and overall survival of human HCC patients. **(D)** Real-time PCR analysis of *Barx1* expression in normal liver (n = 10), 40 pairs of HCCs and adjacent non-tumorous tissues. **(E)** Relative mRNA expression of *Barx1* in HCC patient samples with recurrence (n = 10) or without recurrence (n = 10). **(F)** Relative mRNA expression of *Barx1* in HCC patient samples with metastasis (n = 30) or without metastasis (n = 30). **(G)** Real-time PCR analysis of *Barx1* mRNA levels in primary HCC tissues and paired metastatic HCC tissues (n=10). *P < 0.05.

To explore the fucntion of Barx1 in determining the clinical outcomes of HCC patients, we examined its expression in 315 HCC patients with a tissue microarray. Our immunohistochemistry results show that Barx1 protein levels are significantly decreased in human HCC tissues than adjacent non-tumorous tissues (Figure 1A and 1B). Low expression of Barx1 is significantly correlated with multiple tumor numbers, maximum tumor size, poor tumor differentiation, and a higher tumor-nodule-metastasis (TNM) stage (Table 1). We performed the Kaplan-Meier analysis and found that patients with low expression of Barx1 has shorter overall survival rates than patients with high expression of Barx1 (Figure 1C). Using a multivariate Cox proportional hazards model, we found that low expression of Barx1is an independent and significant predictor for reduced survival (P=0.007) after curative resection (Table 2).

**Table 1:** Correlation between Barx1, MMP9 and MGAT5 expression and clinicopathological characteristics in 315 HCC tissues

		Tumor Barx1 expression			Tumor MMP9 expression			Tumor MGAT5 expression		
		Low	High	P Value	Low	High	P Value	Low	High	P Value
		(n=198)	(n=117)		(n=133)	(n=182)		(n=119)	(n=196)	
Age(year)	<60	128	80	0.499	93	115	0.212	85	123	0.115
	≥60	70	37		40	67		34	73	
Sex	Female	29	23	0.247	27	25	0.121	25	27	0.094
	Male	169	94		106	157		94	169	
Serum AFP(ng/ml)	<20	52	33	0.707	40	45	0.291	37	48	0.201
	≥20	146	84		93	137		82	148	
HBV	Yes	173	101	0.789	118	156	0.433	107	167	0.228
	No	25	16		15	26		12	29	
Cirrhosis	Present	168	104	0.313	113	159	0.54	100	172	0.351
	Absent	30	13		20	23		19	24	
Tumor No.	Single	172	98	0.023*	103	157	0.256	91	169	0.027*
	Multiple	26	29		30	25		28	27	
Tumor Size(cm)	<5	59	53	0.005*	58	54	0.011*	59	53	0.000*
	≥5	139	64		75	128		60	143	
Tumor differentiation	I-II	139	102	0.001*	90	151	0.002*	103	138	0.001*
	III-IV	59	15		43	31		16	58	
TNM stage	I-II	61	59	0.001*	41	79	0.023*	58	62	0.002*
	III	137	58		92	103		61	134	

Abbreviation: AFP, αlpha-fetoprotein; HBV, hepatitis B virus

*P<0.05.

**Table 2:** Univariate and multivariate analysis of factors associated with survival of 315 HCC patients

	Overall survival					
	Univariate analysis			Multivariate analysis		
Variables	HR	95%CI	P Value	HR	95%CI	P Value
Age(year) (≥60 vs <60)	0.8958	0.6580–1.219	0.4871			
Sex(female vs male)	0.6165	0.4450-0.9740	0.0521			
Serum AFP(ng/ml) (≥20 vs <20)	1.167	0.8392-1.615	0.3671			
HBV (no vs yes)	0.8942	0.5668-1.391	0.6071			
Cirrhosis (present vs absent)	1.556	0.9808-2.204	0.0643			
Tumor Number (single vs multiple)	0.5553	0.4206-0.8662	0.0069	0.743	0.465-1.188	0.215
Tumor size(cm)(≥5 vs <5)	1.987	1.454-2.640	< 0.0001	1.751	1.207-2.540	0.003
Tumor differentiation (I-II vs III-IV)	1.856	1.455-3.118	0.0001	1.432	1.012-2.025	0.043
TNM Stage (I-II vs III)	1.662	1.215-2.207	0.0014	1.448	1.027-2.040	0.034
Barx2 expression (low vs high)	2.049	1.412-2.596	< 0.0001*	1.659	1.148-2.398	0.007*

Abbreviations: HR:hazard ratio; CI:confidence interval; AFP:alpha-fetoprotein

*P<0.05.

In 40 paired HCC tissues, the mRNA levels of *Barx1* are significantly decreased in HCC tissues as compared to adjacent non-tumor tissues and normal liver tissues (Figure 1D). Second, patients with recurrence of HCC have lower *Barx1* mRNA expression than patients without recurrence (Figure 1E). Third, *Barx1* mRNA expression is much lower in primary HCC tissues from patients who developed metastasis than in primary HCC tissues from patients who did not (Figure 1F). In primary and metastatic HCC for 10 pairs of specimens, *Barx1* mRNA expression is much lower in the metastatic versus primary HCC tissues (Figure 1G). Taken together, these results indicate that down-regulation of Barx1 may facilitate HCC progression and metastasis.

### The increase and decrease of Barx1 levels have opposite impact on HCC invasion and metastasis

**Figure 2:**
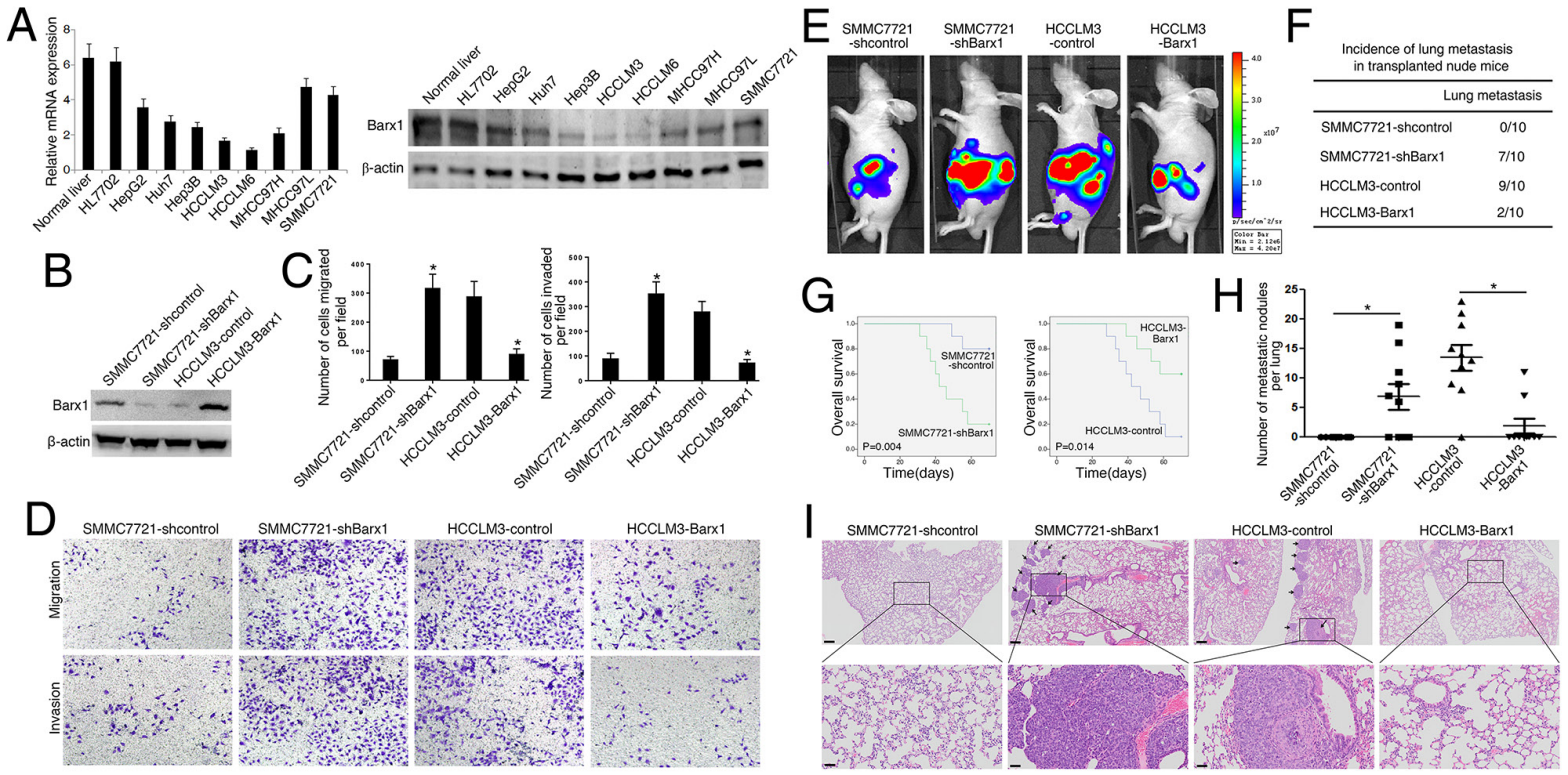
Barx1 inhibits HCC invasion and metastasis **(A)** Real-time PCR and Western blotting analysis of Barx1 expression in different HCC cell lines. **(B)** Western blotting analysis of Barx1 expression in the indicated HCC cells. **(C)** Transwell assay analysis of the migration and invasion abilities of the indicated HCC cells. Data are represented as mean ± SEM for triplicate experiments. **(D)** Representative images of migration and invasion of the indicated cell lines. **(E-I)** *In vivo* metastasis assays. Four stable cell lines were transplanted into the livers of nude mice. (E) Representative bioluminescent imaging (BLI) of the different groups is shown at 10 weeks following orthotopic implantation. The incidence of lung metastasis (F), overall survival (G), the number of lung metastatic foci (H), and representative H&E staining of lung tissues (I) from the different groups is shown. Scale bars represent 200 μm (low magnification) and 50 μm (high magnification). Data are represented as mean ± SEM. *P < 0.05.

We first examined the mRNA and protein levels of Barx1. Our real-time PCR and Western blotting reults show that Barx1 expression is much lower in HCC cells with high metastatic capabilities than in HCC cells with low metastatic capabilities (Figure 2A). To explore the function of Barx1 in HCC migration and invasion, we performed shRNA knockdown or overexpression of Barx1 in SMMC7721 and HCCLM3 cells. Both the increase and decrease of Barx1 expression were confirmed by western blotting analysis (Figure 2B). Our transwell assay results show that decrease of Barx1 protein level significantly increases the migration and invasion capacities of SMMC7721 cells (low metastatic potential), whereas overexpression of Barx1 in HCCLM3 cells significantly reduces cell migration and invasion (Figure 2C and 2D)

We monitored tumor metastasis in mice with an imaging system that detected the luciferase signal. Representative bioluminescent imaging (BLI) of the different groups is shown in Figure 2E. Our histological analysis results shows that the incidence of lung metastasis in the SMMC7721-shBarx1 group is significantly higher than the control group (70% versus 0%), suggesting that decrease of Barx1 protein level promotes HCC metastasis. In the HCCLM3-control group, 9 mice develop lung metastasis; however, only 2 mice develop lung metastasis in the HCCLM3-Barx1 group (90% versus 20%, respectively), indicating that over-expression of Barx1 inhibits HCC metastasis (Figure 2F and 2I). Furthermore, the SMMC7721-shBarx1 group has a shorter overall survival time compared with the SMMC7721-shcontrol group. In contrast, the HCCLM3-Barx1 group displays a longer overall survival time compared with the HCCLM3-control group (Figure 2G). In addition, the number of metastatic lung nodules in the SMMC7721-Barx1 group is significantly higher than the SMMC7721-control group. However, the number of metastatic lung nodules in the HCCLM3-Barx1 group is much lower than the HCCLM3-control group (Figure 2H). Together, these results strongly suggest that Barx1 inhibits HCC invasion and metastasis.

### Barx1 inhibits *MGAT5* and *MMP9* transcription

To explore the molecular mechanisms through which Barx1 regulates HCC metastasis, we compared the mRNA expression profile of SMMC7721-shBarx1 cells with that of SMMC7721-shcontrol cells with a human RT^2^ Profiler PCR Array containing 84 metatasis-related genes. Knockdown of Barx1 upregulates the expression of several metastasis-related genes (Supplementary Table 1), including *MGAT5*, *MMP9*, *MTA1*, *MMP2*, *VEGFA*, *CTSK*, and *CHD4*. Among these genes, *MGAT5* and *MMP9* are the most up-regulated genes upon the depletion of Barx1.

**Figure 3:**
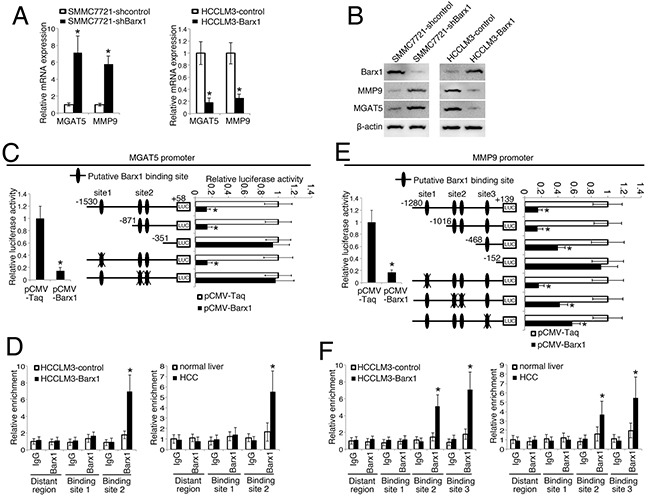
Barx1 inhibits *MGAT5* and *MMP9* transcription **(A and B)** Barx1inhibits MGAT5 and MMP9 expression. After SMMC7721 and HCCLM3 cells were infected with Lenti-shBarx1 or Lenti-Barx1, respectively, the mRNA (A) and protein levels (B) of MGAT5 and MMP9 were detected using real-time PCR and western blot techniques, respectively. **(C and E)** Barx1 inhibits *MGAT5* and *MMP9* transcription. The *MGAT5* or *MMP9* promoter constructs were co-transfected with pCMV-Barx1, and the relative luciferase activity was determined. Deletion and selective mutation analysis identifies Barx1-responsive regions in the *MGAT5* and *MMP9* promoter. Serially truncated and mutated *MGAT5* or *MMP9* promoter constructs were co-transfected with pCMV-Barx1, and the relative luciferase activity was determined. **(D and F)** (Left panel) A ChIP assay demonstrate the direct binding of Barx1 to the *MGAT5* (D) and *MMP9* (F) promoter in HCC cells. Real-time PCR was performed to detect the amounts of immunoprecipitated products. Data are represented as mean ± SEM for triplicate experiments. (Right panel) Barx1 directly binds to the *MGAT5* (D) and MMP9 (F) promoter in primary HCC tissues. The hepatocytes were separated from the primary HCC tissues (n = 6) and normal liver tissues (n = 3). The cells were crosslinked and the chromatin were immunoprecipitated by anti-Barx1 or control IgG antibodies, respectively. Data are represented as mean ± SEM. The y-axis represents the relative enrichment of Barx1 compared to the IgG control. *P < 0.05.

Interestingly, knockdown of Barx1 up-regulates MGAT5 and MMP9 expression in SMMC7721 cells, whereas over-expression of Barx1 decreases MGAT5 and MMP9 expression in HCCLM3 cells (Figure 3A and 3B). The luciferase reporter assay show that Barx1 inhibits *MGAT5* and *MMP9* promoter activities (Figure 3C and 3E). We found three putative Barx1 binding sites in the *MGAT5* promoter. To characterize the roles of these Barx1 binding site in response to Barx1 protein level changes, we generated a series of reporters containing serial 5′ deletions of the *MGAT5* promoter. These constructs were co-transfected with pCMV-Barx1 into target cells. The luciferase reporter assay results indicates that the DNA fragment located between nt −871 and −351 is critical for the transcriptional repression mediated by Barx1. There are two overlapped Barx1-binding sites in this region and simultaneous mutation of both sites significantly reduces the Barx1-mediated repression of the *MGAT5* promoter (Figure 3C). Similarly, two Barx1 binding sites in the *MMP9* promoter are also critical for Barx1-mediated repression (Figure 3E). Our chromatin immunoprecipitation (ChIP) assay results confirm the direct binding of Barx1 protein to the Barx1 binding sites within both the *MGAT5* and *MMP9* promoters in HCC cells and human HCC tissues (Figure 3D and 3F). Taken together, these results indicate that *MGAT5* and *MMP9* are direct target genes of Barx1.

### Barx1 inhibits HCC invasion and metastasis through inhibiting MGAT5 and MMP9 expression

**Figure 4:**
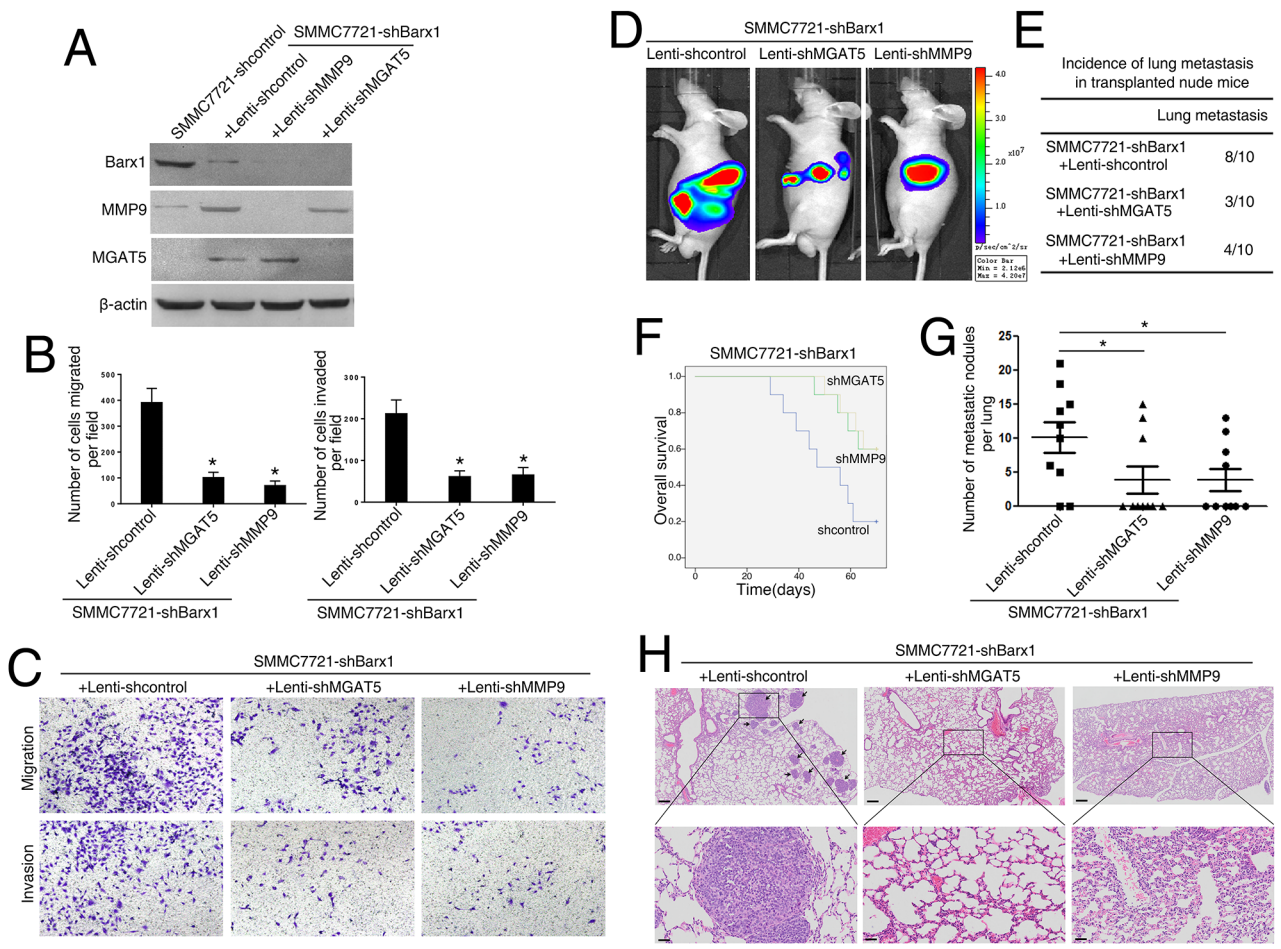
Barx1 inhibits HCC metastasis through the inhibition of MGAT5 and MMP9 expression **(A and B)** Following the infection of the SMMC7721-shBarx1 cells with the lentivirus Lenti-shMGAT5 or Lenti-shMMP9, respectively, (A) the protein levels of Barx1, MGAT5, and MMP9 were detected by western blot, and (B) the cell migration and invasion capacities were assessed using transwell assays. Data are represented as mean ± SEM for triplicate experiments. **(C)** Representative images of migration and invasion of the indicated cell lines. **(D-H)** *In vivo* metastatic assay. Cell lines were transplanted into the livers of nude mice. (D) Representative bioluminescent imaging (BLI) of the different groups is shown at 10 weeks following orthotopic implantation. The incidence of lung metastasis (E), overall survival (F), the number of lung metastatic foci (G), and representative H&E staining of lung tissues (H) from the different groups is shown. Scale bars represent 200 μm (low magnification) and 50 μm (high magnification). Data are represented as mean ± SEM. *P < 0.05.

To study whether MGAT5 and MMP9 are required for Barx1-mediated cancer cell invasion and metastasis, we reduced the expression of MGAT5 and MMP9 in SMMC7721-shBarx1 cells with shRNA knockdown (Figure 4A). The depletion of both MGAT5 and MMP9 significantly decreases the migration and invasion abilities induced by Barx1 knockdown (Figure 4B and 4C).

Our *in vivo* metastatic assay results show that 8 mice develop lung metastasis in the control group (SMMC7721-shBarx1 + Lenti-shcontrol). However, in the MGAT5 and MMP9 inhibition groups (SMMC7721-shBarx1 + Lenti-shMGAT5; SMMC7721-shBarx1 + Lenti-shMMP9), only 3 and 4 cases develop lung metastasis (Figure 4D, 4E, and 4H; and Supplementary Figure 1), as confirmed by both BLI and histological analysis. Moreover, the MGAT5 and MMP9 inhibition groups have a longer overall survival time compared with the control group (Figure 4F). In addition, the number of metastatic lung nodules in the MGAT5 and MMP9 knockdown groups are much lower than the control group (Figure 4G). These results indicate that MGAT5 and MMP9 are required in Barx1-mediated HCC invasion and metastasis.

### Barx1 protein levels inversely correlates with MGAT5 and MMP9 expression in human HCC

**Figure 5:**
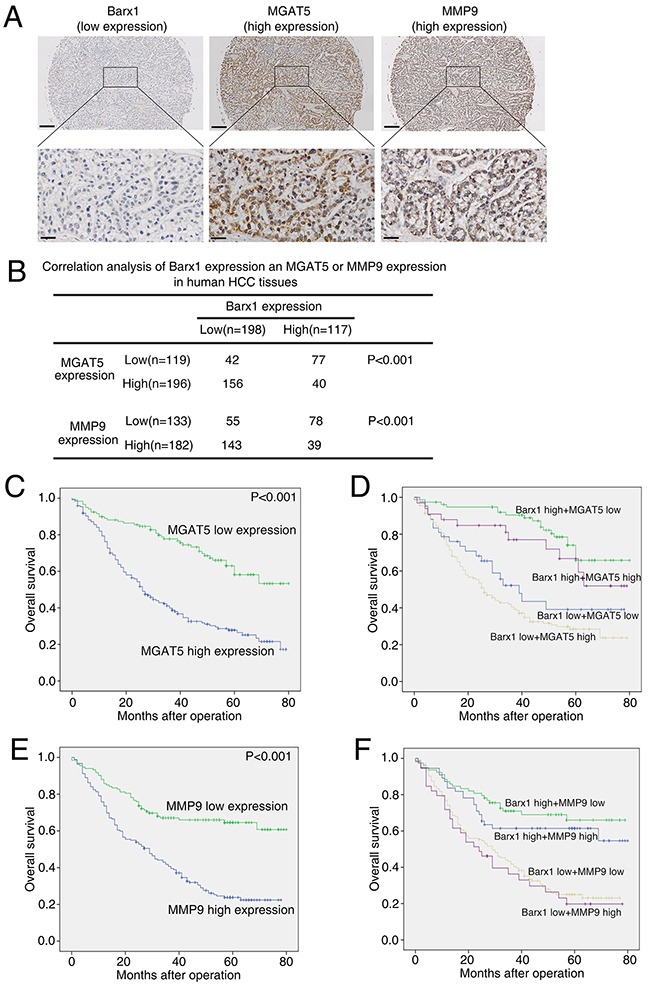
Barx1 expression is inversely correlated with MGAT5 and MMP9 expression in human HCC tissues **(A)** Representative immunohistochemical images of Barx1, MGAT5, and MMP9 in HCC tissues. Scale bars represent 200 μm (low magnification) and 50 μm (high magnification). **(B)** The association between the expression of Barx1 and either MGAT5 or MMP9 in HCC patients. **(C and E)** Kaplan-Meier analysis of the correlation between MGAT5 or MMP9 expression and overall survival of human HCC patients. **(D and F)** Kaplan-Meier analysis of concurrent Barx1 and MGAT5 or MMP9 expression with overall survival.

We further examined the relationship between Barx1 protein level and MGAT5 or MMP9 expression levels in human HCC tissues. Both over-expression of MGAT5 and MMP9 strongly correlates with poorer tumor differentiation, higher TNM stage (Table 1), and poorer survival (Figure 5C and 5E). Our immunohistochemistry assay results show that the level of Barx1 protein inversely correlates with MGAT5 and MMP9 expression (Figure 5A and 5B). Patients were divided into four groups based on the protein level of Barx1 and either MGAT5 or MMP9 expression. The results of our Kaplan-Meier analysis show that patients with low levels of Barx1 protein and high expression of MGAT5 have the shortest overall survival (Figure 5D). Similarly, patients with low levels of Barx1 protein and high expression of MMP9 also have the shortest overall survival (Figure 5F).

## DISCUSSION

Metastasis is the hallmark of human cancer [16]. Recurrence and metastasis are the two major causes of patient mortality in human HCC after surgical resection [17, 18]. Exploration of cancer-specific factors and their regulatory signaling contributes to the identification of novel biomarkers and theraputic targets for human HCC. In this study, we observed that the expression of Barx1 is significantly downregulated in HCC tissues than adjacent nontumor tissues and normal liver. Low expression of Barx1 positively correlates with aggressive tumor progression and poor survival. Multivariate analysis indicates that the level of Barx1 protein is an independent and significant biomarker for HCC survival. We also found that the level of Barx1 protein is much lower in HCC tissues from patients who developed metastasis than those from patients who did not. These evidences indicate that loss of Barx1 may facilitate HCC progression and metastasis.

Loss of homeobox-containing genes have been reported to contribute to cancer proliferation, apoptosis, differentiation, adhesion, and migration [19–23]. For example, loss of Barx2 promotes the malignant differentiation of medullary thyroid carcinoma and is associated with poor prognosis [24]. Low level of Barx2 protein has a negative correlation with HCC clinical outcomes and over-expression of Barx2 inhibits HCC metastasis [11]. Both Barx1 and Barx2 contain a glutamine residue in the third helix of the homeodomain, which confers a binding property for targeting core sequence of TAAT that are present in the promoters of several cell adhesion molecules [7, 25]. In this study, we found that depletion of Barx1 stimultates the invasion and metastasis of SMMC7721 cells, whereas ectopic over-expression of Barx1 inhibits the invasion and metastasis of HCCLM3 cells. These results suggest that down-regulation of Barx1 promotes HCC invasion and metastais.

Metastasis is a multi-step biological process which involves the escape of tumor cells from a primary tumor, local invasion of the surrounding tissue, entrance into the circulation system, extravasation into the parenchyma of distant tissues and ultimately the establishment of secondary tumors [26]. Using metatasis PCR Aarray, we found that down-regulation of Barx1 reactivates many metastatic related genes. Among these genes, *MGAT5* and *MMP9* are the most reactivated genes. To migration and invasion, cancer cells must go through a multi-stage of intracellular changes such as rearrangement of cytoskeletal structure, cell–matrix interaction and remodeling of extracellular matrix (ECM) [16]. Matrix metalloproteinases are critical for cancer cell invasion and dissemination [27]. For example, MMP-9 could not only destroy type IV collagen, which lead to the degradation of ECM proteins, but also contribute to the formation of metastatic lesion sites in remote organs [28, 29]. High expression of MMP-9 indicates poor prognosis of HCC [30, 31]. *MGAT5* gene encodes an enzyme named N-acetylglucosaminyltransferase V that catalyzes the formation of β1, 6-branched N-glycans, which strongly associates with cancer metastasis [32–34]. Previous studies reported that MGAT5 stabilizes matriptase enzyme and promotes epithelial-to-mesenchymal transition process [35, 36]. Over-expression of MGAT5 promotes HCC malignant progression [37, 38]. These studies suggest that MGAT5 and MMP9 are critical regulator of cancer metastasis. In this study, we found that *MGAT5* and *MMP9* are direct transcriptional targets of Barx1. Barx1 inhibits their transcription through directly binding to their promoters. Knockdown of MGAT5 and MMP9 decreases the migration, invasion and metastasis induced by Barx1 depletion. Furthermore, the protein level of Barx1 negatively correlates with MGAT5 and MMP9 expression in human HCC tissues. Patients with low expression of Barx1 and high expression of MGAT5 or MMP9 have the lowest overall survival times. Taken together, our data indicate that decrease of Barx1 protein level facilitates HCC invasion and metastasis through upregulating MGAT5 and MMP9 expression.

In summary, we report a novel tumor suppressor function of Barx1, which is frequently down-regulated in human HCC tissues. Barx1 blocks HCC invasion and metastasis through the down-regulation of MGAT5 and MMP9 expression. Thus, loss of Barx1 represents a prognostic biomarker for human HCC.

## MATERIALS AND METHODS

### Cell culture

Human HCC cells (HepG2, Huh-7, and Hep3B) were purchased from the American Type Culture Collection. Human HCC cells (SMMC7721, MHCC97L, MHCC97H, HCCLM3, and HCCLM6) were kindly provided by Dr. Tang ZY (Liver Cancer Institute, Zhongshan Hospital, Fudan University, Shanghai, China). MHCC97L, MHCC97H, HCCLM3, and HCCLM6 cells are stepwise potentially metastatic cell lines with the same genetic background but different lung metastatic potentials. Immortalized liver cell line (HL-7702) was purchased from the Institute of Biochemistry and Cell Biology, Chinese Academy of Science, China. Cells were cultured in Dulbecco's Modified Eagle Medium (DMEM) at 37°C in a 5% CO_2_ incubator. The medium was supplemented with 10% FBS, 100 μg/ml penicillin, and 100 μg/ml streptomycin.

### Patients and follow-up

Four commercial HCC tissue microarray chips containing 315 primary HCC samples with complete clinical and pathological data were purchased from Shanghai Outdo Biotech CO., ltd (Shanghai, China). These samples were used for immunohistochemical staining (IHC). The whole cohort consisted of 263 (83.5%) men and 52 (16.5%) women. The mean age of patients in this sample group was 55.30 years (range from 25 to 80). Diagnosis was confirmed by at least two pathologists and the histology and clinical stages were classified according to the guidelines of NCCN2010. Overall survival (OS) and disease-free survival (DFS) rates were defined as the interval between initial surgery and clinically or radiologically determined recurrence/metastasis and death, respectively. In addition, 40 pairs of frozen fresh tumor liver tissues and peripheral nontumor tissues from the Xijing hospital were collected after surgical resection and stored in liquid nitrogen. These tissues were used to detect the mRNA levels of Barx1. This study was approved by the Ethics Committee of the Fourth Military Medical University.

### Immunohistochemical (IHC) staining

Briefly, after baking on a panel at 60°C for 1 hr, the tissue sections were deparaffinized with xylene and rehydrated through gradient ethanol immersion. Endogenous peroxidase activity was quenched by 3% (vol/vol) hydrogen peroxide in methanol for 12 min, followed by three 3-min washes with phosphate-buffered saline (PBS). Then the slides were immersed in 0.01 mol/L citrate buffer solution (pH 6.0) and placed in a microwave oven for 30 min. After washing in PBS (pH 7.4, 0.01 mol/L), the sections were incubated in a moist chamber at 4°C overnight with the primary antibody diluted in PBS containing 1% (wt/vol) bovine serum albumin. The primary antibodies are as follows: Barx1 (Santa Cruz, sc-81956), MMP9 (Sigma, HPA001238), and MGAT5 (Novusbio, NBP1-83354). Negative controls were performed by replacing the primary antibody with preimmune mouse serum. After three 5 min washes with PBS, the sections were treated with a peroxidase-conjugated second antibody (Dako Cytomation, Glostrup, Denmark) for 30 min at room temperature, followed by additional three 5 min washes with PBS. Reaction product was visualized with diaminobenzidine for 2 min. Images were obtained under a light microscope (Olympus, Japan) equipped with a DP70 digital camera.

Analysis was performed by two independent observers who were blinded to the clinical outcome. The immunostaining intensity was scored on a scale of 0 to 3: 0 (negative), 1 (weak), 2 (medium) or 3 (strong). The percentage of positive cells was evaluated on a scale of 0 to 4: 0 (negative), 1 (1%-25%), 2 (26%-50%), 3 (51%-75%), or 4 (76%-100%). The final immuno-activity scores were calculated by multiplying the above two scores, resulting an overall scores which range from 0∼12. Each case was ultimately considered “Low” if the final score ranges from 0∼3, and “High” if the final score ranges from 4∼12.

### Plasmid construction

Plasmid construction was performed according to standard procedures as outlined in our previous study [39]. The primers are presented in Supplementary Table 2. For example, the *MGAT5* promoter construct, (−1530/+58)MGAT5, was generated from human genomic DNA. This construct corresponds to the sequence from −1530 to +58 (relative to the transcriptional start site) of the 5′-flanking region of the human *MGAT5* gene. It was generated with forward and reverse primers incorporating *KpnI* and *XhoI* sites at the 5′ and 3′-ends, respectively. The polymerase chain reaction (PCR) product was cloned into the *KpnI* and *XhoI* sites of the pGL3-Basic vector (Promega). The 5′-flanking deletion constructs of the *MGAT5* promoter, ((−1530/+58)MGAT5, (−871/+58)MGAT5, and (−351/+58)MGAT5), were similarly generated using the (−1530/+58)MGAT5 construct as the template. The Barx1 binding sites in the *MGAT5* promoter were mutated using the QuikChange II Site-Directed Mutagenesis Kit (Stratagene). The constructs were confirmed by DNA sequencing. Other promoter constructs were cloned in the same manner.

### Construction of lentivirus and stable cell lines

Based on the Barx1 sequence (NM_003658.4), 3 short hairpin RNAs (shRNAs) were designed using the siRNA Target Finder (InvivoGen): shBarx1-1, 5′-GCTGGAGAAACGCTTCGAGAA-3′;shBarx1-2,5′-GAGCCAGTTACAGGTGAAGAC-3′;and shBarx1-3,5′-GCCAGTTACAGGTGAAGACGT-3′. Lentiviral vectors encoding shRNAs were generated using PLKO.1-TRC (Addgene) and designated as LV-shBarx1-1, LV-shBarx1-2, LV-shBarx1-3, and LV-shcontrol. shRNA lentiviral transduction particles targeting MMP9 (TRCN0000051438) and MGAT5 (TRCN0000436389) were purchased from Sigma. Lentiviral vectors encoding the human Barx1 gene were constructed in FUW-teto (Addgene) and designated asLV-Barx1. An empty vector was used as the negative control and was designated asLV-control. The lentiviral vectors were transfected into the HCC cells with a multiplicity of infection (MOI) ranging from 40 to 50 in the presence of polybrene (6μg/ml). At 48 hr after transfection, 2.5 μg/ml puromycin (OriGene) was added, and the cells were incubated for 2 weeks to select for transfected cells.

### Quantitative real-time PCR

Total RNA was extracted using TRIzol Reagent (Invitrogen), and reverse ranscription was performed using the Advantage RT-for-PCR Kit (Takara) according to the manufacturer's instructions. For the real-time PCR analysis, aliquots of double-stranded cDNA were amplified using a SYBR Green PCR Kit (Applied Biosystems). The cycling parameters were as follows: 95°C for 15 s, 55-60°C for 15 s, and 72°C for 15 s for 45 cycles. A melting curve analysis was then performed. The Ct was measured during the exponential amplification phase, and the amplification plots were analyzed using SDS 1.9.1 software (Applied Biosystems). For the cell lines, the relative expression levels (defined as the fold change) of the target genes were determined by the following equation: 2^−ΔΔCt^ (ΔCt = ΔCt^target^ – ΔCt^GAPDH^; ΔΔCt = ΔCt^expressing vector^ – ΔCt^control vector^). The expression level was normalized to the fold change that was detected in the corresponding control cells, which was defined as 1.0. For the clinical tissue samples, the fold change of the target gene was determined by the following equation: 2^−ΔΔCt^ (ΔΔCt = ΔCt^tumor^ – ΔCt^nontumor^). This value was normalized to the average fold change in the normal liver tissues, which was defined as 1.0. All reactions were performed in duplicate. The primers used for the real-time PCR were: Barx1, Forward 5′-AATGCAACATCCTTTGGAGATT-3′ and reverse 5′-ATCCCGTTTATTCCTCTTGGTT-3′ [40]; MMP-9, forward 5′-GTGCTGGGCTGCTTTGCTG-3′ and reverse 5′-GTCGCCCTCAAAGGTTTGGAAT-3′; MGAT5, forward 5′-GCTGCCCAACTGTAGGAGAC-3′ and reverse 5′-GAATCAAGGACTCGGAGCAT-3′; GAPDH, forward 5′-GGGAAGGTGAAGGTCGGAGT-3′ and reverse 5′-GGGGTCATTGATGGCAAC-3′.

### Western blot analysis

Tissue and cell lysates were extracted using RIPA lysis buffer with the protease inhibitor phenylmethane sulfonyl fluoride (Merck Millipore, USA). Protein concentration was measured using the BCA protein assay kit (Beyotime Biotechnology) according to the manufacturer's instructions. Equivalent amounts of protein (30μg) were separated on 10% SDS PAGE gel and then transferred onto 0.45μm PVDF membranes (Millipore, Billeria, MA) according to the standard protocols. Membranes were blocked in 5% milk in TBST buffer for 1 hr at room temperature, followed by incubation with primary antibodies at 4°C overnight. After incubation with a secondary antibody for 1 hr at room temperature, proteins were detected using ECL regent (Millipore, Billeria, MA). Primary antibodies were as follows: Barx1 (Santa Cruz, sc-81956), β-actin (sc-47778), MGAT5 (Abcam, ab87977), and MMP9 (Cell Signaling, #13667).

### Luciferase reporter assay

Luciferase activity was detected using the Dual Luciferase Assay (Promega, USA) according to the manufacturer's instructions. The transfected cells were lysed in culture dishes containing a lysis buffer, and the resulting lysates were centrifuged at maximum speed for 1 min in an Eppendorf micro-centrifuge. Relative luciferase activity was determined using a Modulus TM TD20/20 Luminometer (Turner Biosystems, USA), and the transfection efficiencies were normalized according to the Renilla activity.

### Transient transfection

The cells were plated at a density of 1×10^5^ cells/well in a 24-well plate. After 12-24 hr, the cells were co-transfected with 0.6 μg of the expression vector plasmids, 0.18 μg of the promoter reporter plasmids, and 0.02 μg of the pRL-TK plasmids using Lipofectamine 2000 (Invitrogen, USA) according to the manufacturer's instructions. After 5 hr of transfection, the cells were washed and allowed to recover overnight in fresh medium supplemented with 1% FBS for 48 hr. Serum-starved cells were used for the assay.

### Chromatin immunoprecipitation assay (ChIP)

Cells were cross-linked in 1% formaldehyde at 37°C for 10 min. After washing with PBS, the cells were resuspended in 300 μl of lysis buffer. The DNA was sheared to small fragments by sonication. Sonicated chromatin was diluted to a final SDS concentration of 0.1% and aliquots were rotated with antibody O/N at 4°C. The recovered supernatants were incubated with specific antibodies or an isotype control IgG for 2 hr in the presence of herring sperm DNA and Protein A/G Magnetic beads (Thermo Fisher). These antibodies are anti-Barx1 (Santa Cruz, sc-81956) and anti-IgG (Santa Cruz, sc-2025). The immunoprecipitated DNA was retrieved from the beads with 1% SDS and a 1.1 M NaHCO3 solution at 65°C for 6 hr. The DNA was then purified using a PCR Purification Kit (QIAGEN, USA). The primers are shown in Supplementary Table 2.

Fresh frozen tissues were cross-linked in HEPES-formaldehyde solution to a final concentration of 1% formaldehyde at 37°C for 10 min. After washing with PBS, the tissue pellets were resuspended in 800 μl of ChIP lysis buffer (50 mM Tris-Cl pH 8.0, 10 mM EDTA, 1% SDS). Sonicate the tissue on ice. Dilute the sonicated chromatin in RIPA buffer (10 mM Tris-Cl pH 8.0, 1 mM EDTA, 0.5 mM EGTA, 140 mM NaCl, 1% Triton X 100, 0.1% Sodium Deoxycholate, 0.1% SDS) and add to 40 μl packed, pre-washed agarose beads. Incubate for 1 hr with rocking at 4°C. Spin down the pre-cleared chromatin (2000 rpm, 1 min) and transfer the supernatant to a fresh tube. Add the appropriate antibody (5 μg) and incubate at 4°C overnight. After immunoprecipitating the protein-DNA complexes, add the IP mixture to fresh pre-washed agarose beads and incubate for 1.5 hr at 4°C. Spin the IPs at 2000 rpm for 1 min and remove the supernatant. Wash IPs 3 times with 800 μl of RIPA buffer. Add 300 μl of digesting buffer (50 mM Tris-Cl pH 8.0, 1 mM EDTA, 100 mM NaCl, 0.5% SDS) and incubate the tubes at 65°C for 4 hr. Add an equal volume of phenol-chloroform-isoamylalchohol to the tubes, vortex and centrifuge at high speed for 10 min at 4°C. Transfer the aqueous supernatant to a fresh tube and ethanol precipitate the DNA. Centrifuge ethanol-precipitated samples at top speed for 30 min at 4°C and remove the supernatant. Dry the DNA pellet and resuspend in 20 μl of water. Use this DNA for PCR amplification, 2 μl per reaction.

### *In vitro* migration and invasion assays

A 24-well transwell plate (8-mm pore size, Corning, USA) was used to measure each cell line's migratory and invasive ability. For transwell migration assays, 2.5×10^4^ cells were plated in the top chamber lined with a non-coated membrane. For invasion assays, chamber inserts were coated with 200 mg/mL of Matrigel and dried overnight under sterile conditions. Then, 5×10^4^ cells were plated in the top chamber. In both assays, cells were suspended in medium without serum or growth factors, and medium supplemented with serum was used as a chemoattractant in the lower chamber. After incubation at 37°C for 24 hr, the top chambers were wiped with cotton wool to remove the non-migratory or noninvasive cells. The invading cells on the underside of the membrane were fixed in 100% methanol for 10 min, air-dried, stained in 0.1% crystal violet, and counted under a microscope. The mean of triplicate assays for each experimental condition was used.

### *In vivo* metastatic model and bioluminescent imaging

BALB/C nude mice (5 weeks old) were housed under standard conditions and cared for according to the institutional guidelines for animal care. All animal experiments were approved by the Committee on the Use of Live Animals in Teaching and Research (CULATR), Fourth Military Medical University. For the *in vivo* metastasis assays, 4×10^6^ cells in 100 μl of phosphate-buffered saline (PBS) were injected subcutaneously into the flanks of nude mice. After 4 weeks, the subcutaneous tumors were resected and diced into 1-mm^3^ cubes, which were then implanted into the left lobes of the livers of the nude mice (10 per group). For the *in vivo* tracking, different group of cells were infected with firefly luciferase. The *in vivo* tumor formation and metastases were imaged by bioluminescence. D-luciferin (Xenogen, Hopkinton, MA) at 100 mg/kg was injected intraperitoneally into the mice, and bioluminescence was detected using an IVIS 100 Imaging System (Xenogen). After acquiring photographic images of each mouse, luminescent images were captured using various (1-60 seconds) exposure times. The resulting grayscale photographic and pseudo-colored luminescent images were automatically superimposed using the IVIS Living Image (Xenogen) software; this superimposition was performed to facilitate the matching of the observed luciferase signal with its location on the mouse. The survival of the mice was recorded daily. After 10 weeks, the mice were sacrificed, and their lungs were dissected and prepared for standard histological examination.

### Statistical analysis

The quantitative data were compared between groups using the Student's t-test. Categorical data were analyzed using the Fisher's exact test. The cumulative recurrence and survival rates were determined using the Kaplan-Meier method and log-rank test. The Cox proportional hazards model was used to determine the independent factors that influence survival and recurrence based on the variables that had been selected from the univariate analysis. A value of P< 0.05 was considered to be significant. All the analyses were performed using the SPSS software (version16.0).

## SUPPLEMENTARY MATERIALS FIGURE AND TABLES





## Abbreviaions

HCC: hepatocellular carcinoma
TNM: tumor-node-metastasis
ChIP: chromatin immunoprecipitation analysis
Barx1: Barx homeobox1
MGAT5: mannosyl (alpha-1,6-)-glycoprotein beta-1,6-N-acetyl-glucosaminyltransferase 5
MMP9: matrix metallopeptidase 9
