# Supplementary material for: Loss of Barx1 promotes hepatocellular carcinoma metastasis through up-regulating MGAT5 and MMP9 expression and indicates poor prognosis
Source: Oncotarget. 2017 May 30;8(42):71867–80. doi: 10.18632/oncotarget.18288 (PMC5641096; doi:10.18632/oncotarget.18288)
Supplement: Supplementary file 1 [file oncotarget-08-71867-s001.pdf]

Loss of Barx1 promotes hepatocellular carcinoma metastasis through up-regulating MGAT5 and MMP9 expression and indicates poor prognosis

SUPPLEMENTARY MATERIALS

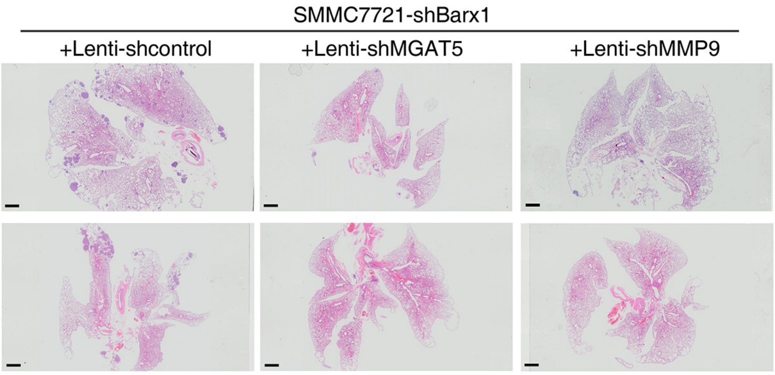

Supplementary Figure 1: Representative HE staining images of the gross observation of lung metastasis from the indicated groups. Scale bars represent 1 mm.

Supplementary Table 1: List of genes differentially expressed in SMMC7721-shBarx1 versus SMMC7721-shcontrol cells using a human metastasis PCR array.

See Supplymentary File 1

Supplementary Table 2: Primer sequences used in the study

Primer name	Primer sequences	Enzyme
Primers for MGAT5 promoter construct:		
(-1530/+58)MGAT5 sense:	5’-TATAGGTACCAGAGTATCTGTTACATAT-3’	KpnI
(-871/+58)MGAT5 sense:	5’-TATAGGTACCTATGCTCAGGTCCTACTT-3’	KpnI
(-351/+58)MGAT5 sense:	5’-TATAGGTACCTGATGGTGATATGACTCC-3’	KpnI
antisense:	5’-TATA_CTCGAGCTGGTCTCACTCTGGCTC-3’	XhoI
Primers for MGAT5 promoter site-directed mutagenesis:		
Barx1 binding site 1 mutation sense:	5’-TTCATGACCCcgcgAGCATATATA-3’	
Barx1 binding site 1 mutation antisense:	5’-TATATATGCTcgcgGGGTCATGAA-3’	
Barx1 binding site 2 mutation sense:	5’-ATCTGGCTGCcgcgATATTGAAAC-3’	
Barx1 binding site 2 mutation antisense:	5’-GTTTCAATATcgcgGCAGCCAGAT-3’	
Primers used for ChIP in the MGAT5 promoter:		
Distant region sense:	5’-TGTAGAGATGCATTCTGT-3’	
Distant region antisense:	5’-CCAGTTCAGAGACATGCA-3’	
Barx1 binding site 1 sense:	5’-TAAGAGGTAGATAGTAAA-3’	
Barx1 binding site 1 antisense:	5’-ACTGATCATCTAGGCATG-3’	
Barx1 binding site 2 sense:	5’-CTATGCTCAGGTCCTACT-3’	
Barx1 binding site 2 antisense:	5’-GCATGCTGTAGCACAAGA-3’	
Primers for MMP9 promoter construct:		
(-1280/+139)MMP9 sense:	5’-TATAGGTACCTGGCATAAGTGTGATAAT-3’	KpnI
(-1016/+139)MMP9 sense:	5’-TATAGGTACCCATGGCTCACTGTATCCT-3’	KpnI
(-468/+139)MMP9 sense:	5’-TATAGGTACCGGATATCTGACCTGGGAG-3’	KpnI
(-152/+139)MMP9 sense:	5’-TATAGGTACCTCTCATGCTGGTGCTGCC-3’	KpnI
antisense:	5’-TATA_CTCGAGGGTGAGATTGGTTCTCAG-3’	XhoI
Primers for MMP9 promoter site-directed mutagenesis:		
Barx1 binding site 1 mutation sense:	5’-GCTCCTATAGcgcgTTTTCCCCCA-3’	
Barx1 binding site 1 mutation antisense:	5’-TGGGGGAAAAcgcgCTATAGGAGC-3’	
Barx1 binding site 2 mutation sense:	5’-GGCCTTAGTAcgcgAAACCAATCA-3’	
Barx1 binding site 2 mutation antisense:	5’-TGATTGGTTTcgcgTACTAAGGCC-3’	

(*Continued*)

Primer name	Primer sequences	Enzyme
Barx1 binding site 3 mutation sense:	5'-CAGGGGGATCcgcgGTTTCAGAAA-3'	
Barx1 binding site 3 mutation antisense:	5'-TTTCTGAAACcgcgGATCCCCCTG-3'	
Primers used for ChIP in the MMP9 promoter:		
Distant region sense:	5'-TAGCATAGCAGTGAACAC-3'	
Distant region antisense:	5'-ATCTCAGCTCTGCCACTG-3'	
Barx1 binding site 1 sense:	5'-TTGGCATAAGTGTGATAA-3'	
Barx1 binding site 1 antisense:	5'-TCTAAGCTGCAGTGTAAC-3'	
Barx1 binding site 2 sense:	5'-GCAGTGACATAATCATGG-3'	
Barx1 binding site 2 antisense:	5'-CACTGTAGGTTGTAAGTC-3'	
Barx1 binding site 3 sense:	5'-AGGATATCTGACCTGGGA-3'	
Barx1 binding site 3 antisense:	5'-CTTGTGGGAACTGTATGA-3'	
