# Supplementary material for: Loss of Barx1 promotes hepatocellular carcinoma metastasis through up-regulating MGAT5 and MMP9 expression and indicates poor prognosis
Source: Oncotarget. 2017 May 30;8(42):71867–80. doi: 10.18632/oncotarget.18288 (PMC5641096; doi:10.18632/oncotarget.18288)
Supplement: Supplementary file 2 [file oncotarget-08-71867-s002.docx]

Supplemental Table S1. List of genes differentially expressed in SMMC7721-shBarx1 versus SMMC7721-shcontrol cells using a human metastasis PCR array

| **Gene name** | **SMMC7721-shBarx1 versus SMMC7721-shcontrol** | **Description** |
| --- | --- | --- |
| MGAT5 | 7.09 | Mannosyl (alpha-1,6-)-glycoprotein beta-1,6-N-acetyl-glucosaminyltransferase |
| MMP9 | 6.08 | Matrix metallopeptidase 9 (gelatinase B, 92kDa gelatinase, 92kDa type IV collagenase) |
| MTA1 | 5.76 | Metastasis associated 1 |
| MMP2 | 4.98 | Matrix metallopeptidase 2 (gelatinase A, 72kDa gelatinase, 72kDa type IV collagenase) |
| VEGFA | 4.48 | Vascular endothelial growth factor A |
| CTSK | 4.48 | Cathepsin K |
| CHD4 | 4.46 | Chromodomain helicase DNA binding protein 4 |
| FN1(fibronectin) | 3.99 | Fibronectin 1 |
| CCL7 | 3.89 | Chemokine (C-C motif) ligand 7 |
| TGFB1 | 3.78 | Transforming growth factor, beta 1 |
| MMP7 | 3.67 | Matrix metallopeptidase 7 (matrilysin, uterine) |
| EPHB2 | 3.45 | EPH receptor B2 |
| CTSL | 3.12 | Cathepsin L1 |
| CXCR2 | 3.09 | Chemokine (C-X-C motif) receptor 2 |
| CXCL12 | 2.99 | Chemokine (C-X-C motif) ligand 12 |
| ETV4 | 2.89 | Ets variant 4 |
| SRC | 2.76 | V-src sarcoma (Schmidt-Ruppin A-2) viral oncogene homolog (avian) |
| TSHR | 2.88 | Thyroid stimulating hormone receptor |
| COL4A2 | 2.28 | Collagen, type IV, alpha 2 |
| CD44 | 2.21 | CD44 molecule (Indian blood group) |
| MMP11 | 2.21 | Matrix metallopeptidase 11 (stromelysin 3) |
| RB1 | 1.99 | Retinoblastoma 1 |
| FGFR4 | 1.98 | Fibroblast growth factor receptor 4 |
| ITGB3 | 1.94 | Integrin, beta 3 (platelet glycoprotein IIIa, antigen CD61) |
| IGF1 | 1.87 | Insulin-like growth factor 1 (somatomedin C) |
| ITGA7 | 1.87 | Integrin, alpha 7 |
| MCAM | 1.81 | Melanoma cell adhesion molecule |
| TRPM1 | 1.71 | Transient receptor potential cation channel, subfamily M, member 1 |
| SMAD4 | 1.71 | SMAD family member 4 |
| CTBP1 | 1.56 | C-terminal binding protein 1 |
| MET | 1.56 | Met proto-oncogene (hepatocyte growth factor receptor) |
| MMP13 | 1.56 | Matrix metallopeptidase 13 (collagenase 3) |
| PLAUR | 1.56 | Plasminogen activator, urokinase receptor |
| SMAD2 | 1.56 | SMAD family member 2 |
| IL18 | 1.38 | Interleukin 18 (interferon-gamma-inducing factor) |
| CXCR4 | 1.29 | Chemokine (C-X-C motif) receptor 4 |
| HGF | 1.24 | Hepatocyte growth factor (hepapoietin A; scatter factor) |
| CDH6 | 1.18 | Cadherin 6, type 2, K-cadherin (fetal kidney) |
| HTATIP2 | 1.13 | HIV-1 Tat interactive protein 2, 30kDa |
| APC | 1.08 | Adenomatous polyposis coli |
| KRAS | 1.08 | V-Ki-ras2 Kirsten rat sarcoma viral oncogene homolog |
| HRAS | 1.07 | V-Ha-ras Harvey rat sarcoma viral oncogene homolog |
| CDKN2A(p16) | 1.04 | Cyclin-dependent kinase inhibitor 2A (melanoma, p16, inhibits CDK4) |
| MYC | 1.03 | V-myc myelocytomatosis viral oncogene homolog (avian) |
| MMP3 | 1.05 | Matrix metallopeptidase 3 (stromelysin 1, progelatinase) |
| CST7 | -1.14 | Cystatin F (leukocystatin) |
| MYCL | -1.19 | V-myc myelocytomatosis viral oncogene homolog 1, lung carcinoma derived (avian) |
| RPSA | -1.19 | Ribosomal protein SA |
| TP53 | -1.31 | Tumor protein p53 |
| TNFSF10 | -1.34 | Tumor necrosis factor (ligand) superfamily, member 10 |
| FLT4 | -1.34 | Fms-related tyrosine kinase 4 |
| NME4 | -1.35 | Non-metastatic cells 4, protein expressed in |
| PNN | -1.38 | Pinin, desmosome associated protein |
| SET | -1.44 | SET nuclear oncogene |
| PTEN | -1.46 | Phosphatase and tensin homolog |
| EWSR1 | -1.56 | Ewing sarcoma breakpoint region 1 |
| NF2 | -1.56 | Neurofibromin 2 (merlin) |
| TCF20 | -1.58 | Transcription factor 20 (AR1) |
| HPSE | -1.65 | Heparanase |
| IL1B | -1.65 | Interleukin 1, beta |
| FXYD5 | -1.67 | FXYD domain containing ion transport regulator 5 |
| RORB | -1.67 | RAR-related orphan receptor B |
| DENR | -1.78 | Density-regulated protein |
| SYK | -1.79 | Spleen tyrosine kinase |
| NME1 | -1.81 | Non-metastatic cells 1, protein (NM23A) expressed in |
| CDH11 | -1.89 | Cadherin 11, type 2, OB-cadherin (osteoblast) |
| GNRH1 | -1.91 | Gonadotropin-releasing hormone 1 (luteinizing-releasing hormone) |
| CD82 | -2.12 | CD82 molecule |
| TIMP3 | -2.19 | TIMP metallopeptidase inhibitor 3 |
| SSTR2 | -2.21 | Somatostatin receptor 2 |
| MDM2 | -2.28 | Mdm2 p53 binding protein homolog (mouse) |
| KISS1 | -2.37 | KiSS-1 metastasis-suppressor |
| SERPINE1 | -2.43 | Serpin peptidase inhibitor, clade E (nexin, plasminogen activator inhibitor type 1), member 1 |
| CTNNA1 | -2.54 | Catenin (cadherin-associated protein), alpha 1, 102kDa |
| TIMP4 | -2.54 | TIMP metallopeptidase inhibitor 4 |
| MMP10 | -2.56 | Matrix metallopeptidase 10 (stromelysin 2) |
| NR4A3 | -2.65 | Nuclear receptor subfamily 4, group A, member 3 |
| KISS1R | -2.67 | KISS1 receptor |
| METAP2 | -2.81 | Methionyl aminopeptidase 2 |
| BRMS1 | -2.98 | Breast cancer metastasis suppressor 1 |
| FAT1 | -2.99 | FAT tumor suppressor homolog 1 (Drosophila) |
| MTSS1 | -3.32 | Metastasis suppressor 1 |
| CDH1(E-cadherin) | -3.39 | Cadherin 1, type 1, E-cadherin (epithelial) |
| TIMP2 | -4.13 | TIMP metallopeptidase inhibitor 2 |
